# Popular Mobile Phone Apps for Diet and Weight Loss: A Content Analysis

**DOI:** 10.2196/mhealth.5406

**Published:** 2016-07-11

**Authors:** Sarah Zaidan, Erin Roehrer

**Affiliations:** ^1^ School of Computing and Information Systems Faculty of Science, Engineering and Technology University of Tasmania Hobart Australia

**Keywords:** applications, diet, monitoring, obesity, weight loss

## Abstract

**Background:**

A review of the literature has revealed that the rates of overweight and obesity have been increasing in Australia over the last two decades and that wellness mobile phone apps play a significant role in monitoring and managing individuals’ weight. Although mobile phone app markets (iTunes and Google Play) list thousands of mobile phone health apps, it is not always clear whether those apps are supported by credible sources. Likewise, despite the prevailing use of mobile phone apps to aid with weight management, the usability features of these apps are not well characterized.

**Objective:**

The research explored how usability taxonomy could inform the popularity of downloaded, socially focused wellness mobile phone apps, in particular weight loss and diet apps. The aim of the study was to investigate the Australian mobile phone app stores (iTunes and Google Play) in order to examine the usability features of the most popular (ie, most downloaded) wellness apps.

**Methods:**

The design of this study comprises 3 main stages: stage 1, identifying apps; stage 2, development of weight loss and diet evaluation framework; and stage 3, application of the evaluation framework. Each stage includes specific data collection, analysis tools, and techniques.

**Results:**

The study has resulted in the development of a justified evaluation framework for weight loss and diet mobile phone apps. Applying the evaluation framework to the identified apps has shown that the most downloaded iTunes and Google Play apps are not necessarily the most usable or effective. In addition, the research found that search algorithms for iTunes and Google Play are biased toward apps’ titles and keywords that do not accurately define the real functionality of the app. Moreover, the study has also analyzed the apps’ user reviews, which served as justification for the developed evaluation framework.

**Conclusions:**

The analysis has shown that *ease of use*, *reminder*, *bar code scanning*, *motivation*, *usable for all*, and *synchronization* are significant attributes that should be included in weight loss and diet mobile phone apps and ultimately in potential weight loss and diet evaluation frameworks.

## Introduction

The World Health Organization (WHO) defines overweight and obesity as abnormal or excessive fat accumulation, which may result in harm to the person’s health [1]. The Centers for Disease Control and Prevention refers to these terms as labels used to describe ranges of weight that are considered greater than the normal healthy weight for a given height [2].

There are many health problems that occur as a consequence of obesity, including musculoskeletal disorders, cardiovascular disease, type 2 diabetes, and some cancers (endometrial, breast, and colon). Many of these diseases are preventable if a person follows a healthy and active lifestyle [1].

According to WHO global estimates [1], in 2014, about 13% of the world’s adult population (11% of men and 15% of women) was obese, while 39% of adults aged 18 years and older (38% of men and 40% of women) were overweight. The rate of obesity has more than doubled between 1980 and 2014 [1]. The main problem is that overweight and obesity is killing more people than underweight worldwide (this includes all people in high-income and most middle-income countries) [1].

One of the most important strategies to manage this problem is behavioral intervention for weight management and lifestyle changes, which requires *self-monitoring*. This strategy not only leads to weight loss but also prevents weight gain or regaining weight and encourages physical activities [3]. Self-monitoring is observing and recording person’s exercise and eating patterns, followed by feedback on those behaviors [3]. Self-monitoring increases self-awareness with regard to targeting behavior and outcomes in relation to food intake goals. In addition, it can act as an early warning system, indicating whether a risk of becoming overweight is increasing [3]. Although health care providers can monitor an individual’s health, they face clear problems with providing treatment and advice to the people who are at risk of overweight and obesity [4]. Self-monitoring combined with healthy weight guidelines provides an alternative solution to weight management [4].

Wellness technologies and wellness apps can be used to monitor users’ health and help them maintain a healthy lifestyle. *Mobile phone apps* play an important role in monitoring and managing individuals’ weight. They provide real-time feedback and can employ persuasive technology among both chronically ill and healthy individuals [5]. Wellness monitoring apps are redefining the concept of self-monitoring by altering the isolated process of self-monitoring into a communal, supportive process whereby multiple individuals with similar health interests can check the user’s progress and give encouraging feedback [5]. MyFitnessPal, Lose it!, FatSecret’s Calorie Counter, and SparkPeople are some of the popular apps with features that can track diets and physical activities [6].

Although mobile app markets (iTunes and Google Play) list hundreds of thousands of health apps, it is not always clear whether those apps are supported by credible sources [7]. Despite the prevailing use of mobile phone apps to aid with weight management, the usability features of these apps are not well characterized [8]. Therefore, the aim of this study was to investigate the Australian mobile phone app stores (iTunes and Google Play) in order to examine the usability features of popular wellness apps, particularly in the area of diet and weight loss. In addition, it aimed to explore and recommend any important aspects of the most popular mobile phone apps for diet and weight loss that could assist users in the selection of diet and weight loss apps and assist developers in decisions regarding those apps.

## Methods

### Research Objectives

In order to achieve the research aims, the following objectives were identified:

1. Identify the most popular weight loss and diet apps according to specific criteria.

2. Build or find a framework for evaluating these apps and apply this evaluation framework to the identified apps.

3. Compare the outcomes of the developed evaluation framework with specific metrics for justification.

### Design

The design of this research has included 3 main stages: stage 1, *identifying apps*; stage 2, *development of the evaluation framework*; and stage 3, *application of the framework*. At the end, a thematic analysis of apps’ user reviews was conducted to serve as justification and exploration step of the results.

#### Stage 1: Identifying Apps

Identifying Apps included a review of the apps that were located in iTunes and Google Play stores. This review was based on predefined inclusion and exclusion criteria. The inclusion criteria for selecting the apps from the stores were as follows: (1) free, no charge, (2) high star rating (1 and 2 considered low ratings, 3 stars standard, and 4 and 5 stars considered high ratings), (3) app language is English, (4) consumer-oriented app, and (5) app should be related to weight loss and diet. The first 2 criteria were considered because they represent important factors for users in terms of selecting mobile phone wellness apps from among alternatives [7]. The third criterion was identified because the study focused on the Australian mobile phone app stores where English was found to be the most commonly spoken language among mobile phone app users [9]. The fourth and fifth criteria were created to serve this study’s purpose. An app was excluded if it did not meet any of these inclusion criteria.

Apps were selected from the stores using the search terms “weight loss” and “diet.” Because of time constraint, only the top 30 popular apps from each store were examined. First, iTunes apps were collected between June 24 and June 25, 2014, using the aforementioned search terms. The Google Play apps were collected using the same search terms from June 26 to June 30, 2014. The list of possible apps for iOS was obtained using iTunes version 11.2 and for Android using Google Play. Second, the 30 most popular iTunes apps were collected on June 25, 2014 and the 30 most popular apps for Google Play on July 4, 2014. As iTunes does not provide an estimate of the number of downloads per app, the displayed search queries on the computer monitor were used as an indication of the apps’ popularity [10]. Therefore, the first 30 displayed apps were considered the 30 most popular iTunes apps. Unlike iTunes, Google Play store provides a worldwide total number of downloads for each app, which has been utilized as a proxy of apps’ popularity. Consequently, the 30 apps that were downloaded the most were considered as the most popular Google Play apps. The 30 most popular iTunes and Google Play apps that resulted from the search terms “diet” and “weight loss” were only included in the analysis of this study.

iTunes and Google Play descriptions were used to review the 5 inclusion criteria per app. The description page of both stores includes a list of features the app offers, user ratings, customer reviews, and 1 to 4 screenshots of what the app looks like when downloaded. The description page provides sufficient information for evaluating the inclusion criteria. Only the apps that met the 5 inclusion criteria were considered; all other apps were excluded.

#### Stage 2: Development of Evaluation Framework

The development of the evaluation framework was achieved using principles of qualitative content analysis to analyze previous studies related to evaluating wellness apps. Qualitative content analysis methods were utilized to draw on existing theories in order to develop the evaluation framework and its elements, which allowed for a meaningful evaluation of the weight loss and diet mobile phone apps.

#### Stage 3: Application of the Framework

The application of the framework started on July 14, 2014. This stage followed the method by Breton et al [11] of applying the evaluation framework to the wellness mobile phone apps. Thus, an Excel worksheet was used to apply the framework to the apps. The application of the evaluation framework required the individual downloading of the refined apps that resulted from stage 1—identifying apps. After the app was downloaded, the framework components were examined. When the app satisfied the framework component, an “X” was assigned as a code to indicate the presence of the framework component (ie, X=1). Otherwise, the cell was left empty. After investigating the 9 components of the framework, the value of the *index score* was calculated. After the framework was applied to all identified apps separately, the apps were ranked according to their index scores. This method of evaluating apps’ content is a valid technique that has been used by other similar app evaluation studies [8,10-12].

#### Thematic Analysis of Apps’ User Reviews

As the third objective of this study was aiming to compare the outcomes of the developed evaluation framework with a specific metric as justification, a deductive thematic analysis was conducted to search for patterns in the apps' user reviews after applying the framework to all apps. The thematic analysis was conducted on August 4, 2014. The most recent user reviews were analyzed for both iTunes and Google Play apps (available user reviews for all months in 2014). The user reviews were analyzed manually by the authors using the deductive way of thematic analysis. Thus, using Excel worksheets, we first coded the reviews, developed themes, and then generated categories. Patterns that were searched for in the users’ reviews were matched either in wording or meaning to the elements of the evaluation framework. In addition, new patterns discovered from the thematic analysis were also considered. There are several studies that demonstrate the usefulness of investigating apps’ user reviews as it can reflect a key part of users' experience [13-16]. Thus, analyzing users’ reviews put the lens on the developed evaluation framework elements. In addition, it allowed for discovering new evaluation elements that should be utilized in future weight loss and diet evaluation frameworks.

## Results

### Stage 1: Identifying Apps

**Figure 1:**
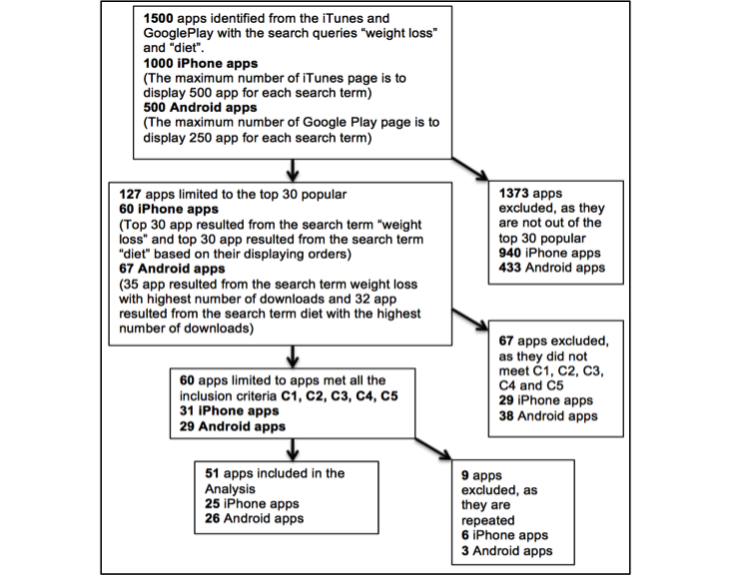
Summary of stage 1 results. (C1 to C5 refer to the 5 inclusion criteria).

Figure 1 summarizes the results of stage 1: identifying apps in the Australian iTunes and Google Play stores.

Some apps in the Australian Google Play market, such as “Woman's DIARY period.diet.cal” developed by HighLab Co Ltd, were excluded from the analysis as they were not related to weight loss or diet. The aforementioned app included a tracking weight feature. However, the content was mainly related to tracking women’s menstrual cycle information. This might suggest that the search engine primarily retrieves apps based on the key terms in the apps’ titles. Some other factors such as the main function of the app must be considered in retrieving apps. Likewise, in the Australian iTunes store, the following 2 apps (FatBooth by PiVi & Co and Fatify - Get fat by Apptly LLC) were retrieved as a result of the following search terms (“weight loss” and “diet”) even though they were not related to diet or weight loss. Apparently, the search engine has considered the term “fat,” hence it retrieved those 2 apps in the search results.

Although this study was performed using the Australian Google Play store, there were many apps retrieved in languages other than English, such as DietShin-diet 청혈주스 레시피. The country of the customer should be considered when retrieving apps. This is another example of search engine bias toward keywords in the apps’ titles.

In Google Play, some apps’ contents were the same; these have been developed by the same developer but appeared under different names. For example, “How To Lose Weight Quickly” and “How To Lose Weight Fast” by Venture Technology Ltd have exactly the same content. One problem with the duplicated apps that appear under different names in the store could be that users might download all of them while they only need to download one. This problem is potentially more serious when the apps are not free. All the aforementioned initial findings of stage 1 indicated that the search algorithm of the stores was biased toward the apps’ titles and keywords and not necessarily reflective of the apps’ content.

### Stage 2: Development of Weight Loss and Diet Evaluation Framework

Analyzing the literature revealed 2 main methods of evaluating mobile phone apps. The first method is the People At the Centre of Mobile Application Development (PACMAD) model by Harrison et al [17]. The PACMAD model includes the following elements: effectiveness, efficiency, satisfaction, learnability, memorability, errors, and cognitive loads. Each of these elements has utility to be evaluated. The PACMAD model considers three factors: user, task, and context [17]. The second method is the method mentioned in the following studies [8,10-12]. The method of these studies evaluated wellness mobile phone apps by first identifying specific usability elements and then evaluating the apps according to the presence or absence of these elements. Each of these studies has its own app scoring system.

The second method was used in this study to evaluate the 51 iTunes and Google Play apps that resulted from stage 1. The second method of evaluating apps was more appropriate for this study as elements of the PACMAD model tend to be general in nature [17]; hence, it might be less efficient if applied to examine the usability of weight loss and diet apps. In addition, applying the first method for large number of apps would exceed the resources available for this study. Thus, the second method of evaluating apps was selected.

The included evaluation elements were related to usability, app design, and index score; these elements resulted from qualitative content analysis of several studies related to evaluating wellness mobile phone apps. The usability elements are concerned with the functionality of the apps. They were based on common evaluation elements gained from behavioral weight loss strategies [18], as well as from the following studies [8,11,12]. However, one of the usability elements (namely, regular physical activity) has been included as it was a common element between some of these studies, which proved the importance of regular physical activity in the context of weight loss and diet. Although it is essential to consider the hedonic design aspects besides the functional side when developing health wellness apps, none of these aforementioned evaluation studies has focused on the apps’ design when evaluating wellness apps. This might be caused by a lack of wellness apps’ design strategies.

Studies in the literature and thus in this field need more investigations in the foreseeable future. Therefore, the authors have decided to consider the design elements in evaluating the selected apps. The design elements were based on wellness apps’ design strategies stated in the study by Alagoz et al [19].

The last element of the developed evaluation framework is the index score. The index score is a score calculated based on the existence of functionality and design elements in the identified apps. Index score values range from 0 (minimum value indicating that the app did not meet any of the developed evaluation elements) to 11 (maximum value indicating that the app incorporated all the developed evaluation elements). The value gained by the index score allowed for an incremental ranking of the apps.

Table 1 lists the elements of the developed weight loss and diet mobile phone apps evaluation framework.

**Table 1:** Elements of the developed weight loss and diet evaluation framework.

Elements			Description
**Usability elements**					
	Self-monitoring	App was scored based on whether or not it provided a means to monitor users’ weight over time.	
	Social support	App was scored on whether it allowed users to access social support services such as message boards, chat rooms, email an expert, or a networking component like Twitter.	
	Knowledge resource	App was scored according to whether it provided a knowledge resource that can increase users’ knowledge/information related to nutrition and awareness of weight control or reduction.	
	Weight loss goal	App was scored on whether it recommends certain weight loss goals for users and whether it allows users to achieve targeted weight.	
	Regular physical activity	The app was scored on whether it recommends a certain amount of physical activity.	
**Design elements**					
	Abstract and reflective	This strategy scores apps on the basis of whether they use a graph, chart, or other virtual means to easily reflect the users’ data.	
	Public	This strategy aims to evaluate an app on whether or not it provided a log-in feature or similar to avoid unwanted disclosure of user’s personal data.	
	Aesthetic	Apps were scored on whether they enable users to customize or adapt some features in the app according to their personal preferences (eg, backgrounds, user interfaces).	
	Controllable	This strategy is concerned with scoring an app on whether it allows a user to manually manage and control access to data. This can remove the possibility of errors that could occur when the apps only depend on sensory data.	
	Trending and historical	This strategy scored an app on whether or not it enables a user to access historical data to show changes and trends over time.	
	Comprehensive	Considers apps that allow users to manually enter data as well as collect sensory data.	
**Index score**		
		A score determined based on the aforementioned 11 functionality and design elements of the developed evaluation framework. The sum of the scores provided a total value to the index score.	

### Stage 3: Application of the Evaluation Framework

None of the iTunes and Google Play apps achieved the highest proposed index score value (ie, 11) after applying the developed evaluation framework to the identified iTunes and Google Play apps. There were 2 interpretations for this outcome:

1. iTunes and Google Play search engines only consider the keywords entered by users to retrieve apps [20-22].

2. The popularity of iTunes and Google Play apps does not necessarily indicate the usability of the apps [20].

**Figure 2:**
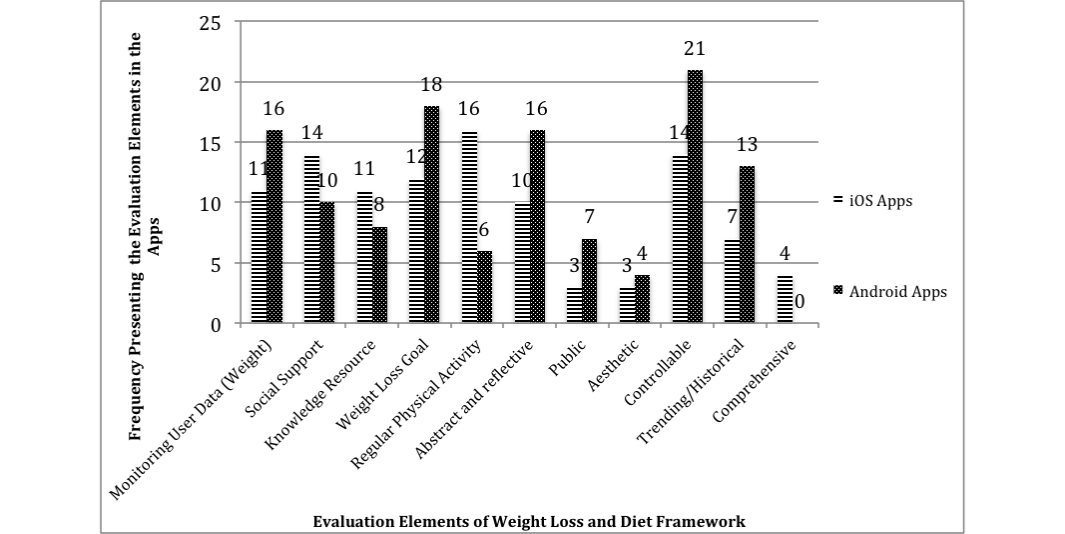
The presence of the evaluation elements in iOS and Android apps.

All the developed evaluation elements were found at least once in iTunes and/or Google Play apps (see Figure 2).

Applying the evaluation framework to the iTunes and Google Play apps showed that 18% (9/51 apps) of the evaluated apps achieved index scores equal to the average index score (ie, 5). Whereas only 37% (19/51) of the evaluated apps achieved index scores that were higher than average, 45% (23/51) achieved index scores that were lower than average. The aforementioned outcome indicates that the proportion of apps that gained index scores below the average is higher than the proportion of apps with index scores above the average.

### Thematic Analysis of Apps’ User Reviews

Many of the 25 identified iTunes apps did not include users' reviews. However, almost all of the 26 Google Play apps had users' reviews. A total of 402 Google Play user reviews and 150 iTunes user reviews were analyzed. Analyzing the iTunes and Google Play apps’ user reviews resulted in 11 categories directly related to the elements of the developed evaluation framework. *Related categories* to elements of the evaluation framework resulted from apps’ user reviews that included themes related in wording or meaning to the elements of the evaluation framework (ie, monitoring user data, social support, knowledge resources, weight loss goal, regular physical activity, abstract and reflective, public, aesthetic, controllable, trending and historical, and comprehensive). As Google Play apps had more user reviews than iTunes apps, a larger number of *related categories* were found when analyzing the Google Play apps’ user reviews.

**Figure 3:**
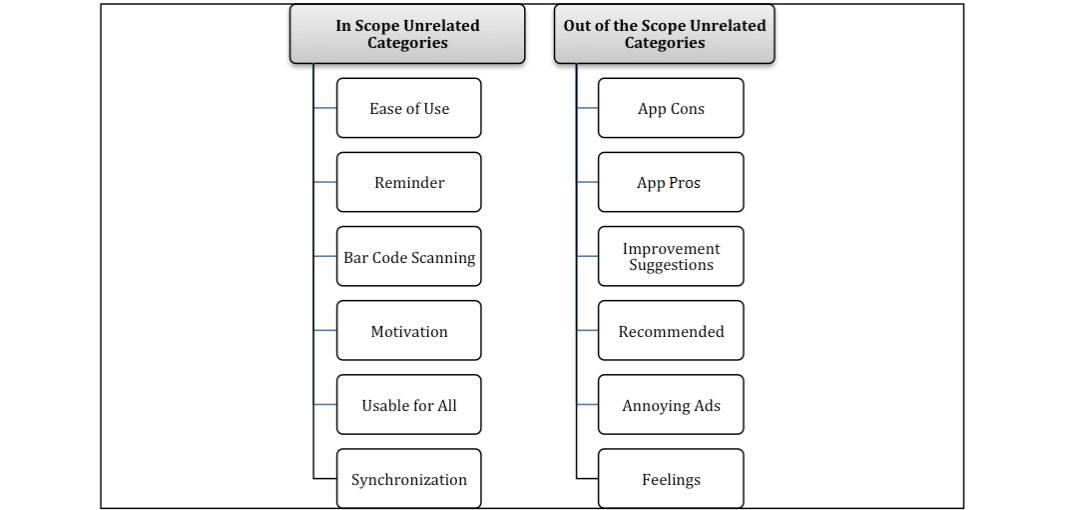
Unrelated categories that resulted from thematic analysis.

Analyzing the iTunes and Google Play apps’ reviews also resulted in 12 categories that were not related to the elements of the evaluation framework. *Unrelated categories* resulted from analyzing users' reviews that included themes not related in wording or meaning to elements of the evaluation framework (see Figure 3). Some of these categories emerged as a result of analyzing iTunes and Google Play users' reviews, while some only resulted from either one of them.

As an additional justification step for the outcomes of the evaluation framework, the emerged *related categories* were compared with the gained index score values. The comparison led to two main conclusions. First, the numbers of *related categories* recognized by analyzing iTunes apps’ user reviews relatively match the index score values obtained by employing the evaluation framework. Second, the numbers of *related categories* identified by analyzing the Google Play users' reviews more closely matched the index score values. As the analysis of the content used a quantitative element (ie, index score) as part of the rationale for inclusion of the ultimate framework elements, accordingly the *related categories* were viewed in the same manner. Conversely, because of the way the *unrelated categories* were formed, the interpretative nature of the study did not compare their existence with the elements of the evaluation framework (ie, index score values). Accordingly, the *unrelated categories* were not ranked or ordered through any type of quantitative measure. This distinction between the analysis of the related and unrelated categories is in line with the philosophical nature of this study, which is subjective ontology and interpretive epistemology.

## Discussion

In stage 1 of this study, the most popular weight loss and diet apps were identified according to specific criteria. The initial findings of stage 1 demonstrated that the search algorithm of iTunes and Google Play was biased toward apps’ titles and keywords. Stage 2 of this study developed a justified weight loss and diet apps’ evaluation framework by utilizing a content analysis of the literature. The developed evaluation framework included elements related to the usability and design of the apps.

Even though the identified apps were the most popular ones on iTunes and Google Play, after applying the developed evaluation framework to the identified apps, none of the apps was able to achieve the maximum index score value. There were two ways to interpret this initial finding. The first interpretation claims that the search algorithm of iTunes and Google Play was biased toward apps’ titles and keywords, which does not necessarily reflect the functionality of the app. Hence, when the developed evaluation framework evaluated these apps, they achieved low index scores. The second interpretation was that the most downloaded iTunes and Google Play apps are not necessarily the most usable and effective apps.

Analyzing the apps’ user reviews has resulted in 11 categories that supported the identified evaluation elements of the developed evaluation framework. The presence of 11 *related categories* in the iTunes and Google Play apps’ user reviews at least once provided additional assurance of the robustness of the developed framework and its outcomes. All the 11 identified thematic categories were related to the identified evaluation elements of the developed framework. This indicated that none of the identified evaluation framework elements was irrelevant or inappropriate for evaluating weight loss and diet apps.

Analyzing the apps’ user reviews resulted in 12 unrelated categories. On one hand, there were six unrelated categories that considered within the scope of this study and hence analyzed in detail (i.e. the *ease of use*, *reminder*, *BCS*, *motivation*, *usable for all*, and *synchronization)* as they were found in the users' reviews of apps that had high index score values. The existence of these categories in the high index score users’ reviews reflected the importance of these elements in health apps, particularly in weight loss and diet apps. On the other hand, the following six unrelated categories that resulted from the thematic analysis of apps users’ reviews namely *app cons*, *app pros*, *improvement suggestions*, *recommended*, *annoying ads*, *feelings* have not been analyzed in detail as some of these categories found in apps users’ reviews of apps with low index score values or they were beyond the scope of this study.

The *ease of use* category was considered one of the most important *unrelated categories* as it was in the user reviews of almost all the apps that gained high index score values. The attribute of easy to use in mobile phone apps encourages people to use wellness apps [7]. One of the factors that affect individual choices of apps is ease of use [23]. Ease of use of health apps was considered one of the important aspects missing from the developed evaluation framework. Therefore, as it was considered one of the factors that encourage use of the wellness app, this element is recommended to be included in any future evaluation frameworks for weight loss and diet apps.

*Reminder*, *BCS*, *motivation*, *usable for all*, and *synchronization* were categories that were not included in the developed evaluation framework even though analysis of the users' reviews revealed their importance. There was agreement on the importance of the *reminder* element in the literature. However, it was not incorporated in the framework, because evaluating this element for all the identified apps would require resources that exceed the scope of this study. Mobile phone health functions such as reminders and alerts could encourage individuals to sustain positive attitudes and improve quality of life among adults [24]. Hence, the inclusion of the *reminder* attribute in future evaluation frameworks of health apps, particularly weight loss and diet apps, seems vital.

Bar code scanning can be used to scan foods to be consumed. In addition, BCS could provide other information such as data concerning exercise [25]. For example, scanning a box of biscuits via an app with BCS feature could reveal that the biscuit contains 140 calories. This calorie data can be transmitted into apps through BCS. Then, the apps could also specify the amount of exercise needed to burn those calories. Therefore, BCS can be used to assist individuals to achieve any weight control goal if added to weight loss and diet apps [26]. The inclusion of the BCS attribute in upcoming weight loss and diet apps and evaluation frameworks is essential.

*Motivation* is an important attribute that should be included in weight loss and diet apps and their evaluation frameworks. Wellness technologies and wellness apps should be designed to motivate users to continue using wellness technology and to achieve their goals [27]. For example, there are several attributes that can increase motivation toward physical activity, such as real-time feedback and having a virtual personal trainer. These attributes would strongly motivate users toward physical activity [27]. In addition, the design of user interface of mobile phone wellness apps can support and motivate users toward initial and long-term use [27]. These motivating attributes were not evaluated for the apps in this study. However, they should be included in future weight loss and diet apps and their evaluation frameworks.

*Usable for all* and *synchronization* elements were not previously mentioned in the analyzed studies that evaluate wellness apps. However, the inclusion of the perception of these categories in future evaluation frameworks for weight loss and diet apps is recommended. *Usable for all* was identified in this study as an app suitable for a wide variety of individuals. For example, wellness apps can be used in different languages; they can include different exercises, and can be suitable for women, men and a wide range of age groups. The app may include different products, diet plans and foods that are suitable for different people such as individuals with diabetes, pregnant women, vegetarian people, etc. By offering several options, the app would not be restricted to specific group of users. Instead, it would give them the freedom of choice. This can be one of the factors that attract users to use the app.

These days, *synchronization* is a very important aspect of mobile phone apps. Mobile phone apps can produce one more function if synchronized with other technology (eg, with wearable medical devices) [28]. Wearable medical devices can provide a huge advantage when it comes to monitoring and early detection of symptoms [28]. The sensors in these wearable medical devices enable monitoring of vital signs and physiological parameters such as electrocardiogram, heart rate, body activity, blood pressure, and weight, to name but a few [28]. Monitoring these features can greatly help the users of weight loss and diet apps. Early examples of independent wearable devices are Fitbit, Jawbone, and Samsung Gear Fit. Currently, Apple has developed a sensor-laden smart watch, and that wearable device synchronizes with an iPhone over Bluetooth and other wireless technologies. Likewise, Google is working on its own smart watch. Similarly, recently Samsung introduced an improved smart watch that supports basic health measurement functionality [29]. Therefore, it can be concluded that the use of these wearable health devices, which enhance and help manage health features, will increase in the future. Hence, the ability of apps to sync with such devices would further inform the evaluation of weight loss and diet apps.

As it has been found that ease of use, reminder, bar code scanning (BCS), motivation, usable for all, and synchronization are significant attributes, it should be incorporated in weight loss and diet mobile phone apps, and thus in potential weight loss and diet evaluation frameworks.

When it comes to selecting weight loss and diet apps from iTunes and Google Play stores, users should be aware that the search engines of these stores are biased toward apps’ titles and keywords, and hence one might end up with some apps that do not really serve the intended weight loss and diet functions. In addition, users should be mindful that the most popular apps are not necessarily the most usable and effective. Therefore, popularity is by no means a measure of usability and effectiveness of apps. Diet and weight loss apps’ users and developers can utilize elements of the developed evaluation framework as a basis for their selection and development of apps based on robust literature.

There was a time constraint on this study (only 6 months), and the qualitative nature of the study ideally requires a longer time span. Thus, the number of apps included in the analysis was reduced. As a result of this, the number of iTunes users' reviews was very small, which led to not-so-fully saturated thematic categories. A longitudinal study or one with more time and resources can include more apps and users' reviews and thus give more accurate results. The limitation of researcher bias can be overcome by applying the evaluation framework more than once to the identified apps. This study merely focused on evaluating the most popular iTunes and Google Play apps. There is here an opportunity for future research to evaluate popular and unpopular apps so as to compare the results. Likewise, as this study has focused only on evaluating high-rated apps, future research could consider the evaluation of both high- and low-rated apps. Such research could determine if there is a relationship between high- and low-rated apps on the one hand and the index score values of evaluated apps on the other. The numbers of analyzed iTunes and Google Play users' reviews were relatively small, so that a greater number of users' reviews may result in additional thematic categories from the analyzed reviews. Future improvement to the evaluation framework can be achieved by adding the identified in-scope *unrelated categories* that resulted from the thematic analysis. This will no doubt result in a more robust and comprehensive weight loss and diet evaluation framework.

## Abbreviations

BCS: bar code scanning
WHO: World Health Organization
